# Ciguatoxin Detection in Flesh and Liver of Relevant Fish Species from the Canary Islands

**DOI:** 10.3390/toxins14010046

**Published:** 2022-01-09

**Authors:** María José Ramos-Sosa, Natalia García-Álvarez, Andres Sanchez-Henao, Freddy Silva Sergent, Daniel Padilla, Pablo Estévez, María José Caballero, José Luís Martín-Barrasa, Ana Gago-Martínez, Jorge Diogène, Fernando Real

**Affiliations:** 1 Division of Fish Health and Pathology, University Institute of Animal Health and Food Safety (IUSA), University of Las Palmas de Gran Canaria, 35416 Arucas, Spain; maria.ramos146@alu.ulpgc.es (M.J.R.-S.); julian.sanchez101@alu.ulpgc.es (A.S.-H.); freddy.silva101@alu.ulpgc.es (F.S.S.); daniel.padilla@ulpgc.es (D.P.); mariajose.caballero@ulpgc.es (M.J.C.); joseluis.martin@ulpgc.es (J.L.M.-B.); fernando.real@ulpgc.es (F.R.); 2 Biomedical Research Center (CINBIO), Analytical and Food Chemistry Department, University of Vigo, Campus Universitario, 36310 Vigo, Spain; paestevez@uvigo.es (P.E.); anagago@uvigo.es (A.G.-M.); 3 Research Unit, Hospital Universitario de Gran Canaria, Dr Negrín, 35019 Las Palmas de Gran Canaria, Spain; 4 Marine and Continental Waters Programme, Institut de Recerca i Tecnologies Agroalimentàries (IRTA), Ctra. Poble Nou, km. 5.5, 43540 Sant Carles de la Ràpita, Spain; jorge.diogene@irta.cat

**Keywords:** ciguatoxins, amberjack, dusky grouper, moray eel, common two-banded seabream, Canary Islands

## Abstract

The Canary Islands are a ciguatoxin (CTX) hotspot with an established official monitoring for the detection of CTX in fish flesh from the authorised points of first sale. Fish caught by recreational fishermen are not officially tested and the consumption of toxic viscera or flesh could lead to ciguatera poisoning (CP). The objectives of this study were to determine the presence of CTX-like toxicity in relevant species from this archipelago, compare CTX levels in liver and flesh and examine possible factors involved in their toxicity. Sixty amberjack (*Seriola* spp.), 27 dusky grouper (*Epinephelus marginatus*), 11 black moray eels (*Muraena helena*) and 11 common two-banded seabream (*Diplodus vulgaris*) were analysed by cell-based assay (CBA) and Caribbean ciguatoxin-1 (C-CTX1) was detected by liquid chromatography mass spectrometry (LC-MS/MS) in all these species. Most of the liver displayed higher CTX levels than flesh and even individuals without detectable CTX in flesh exhibited hepatic toxicity. Black moray eels stand out for the large difference between CTX concentration in both tissues. None of the specimens with non-toxic liver showed toxicity in flesh. This is the first evidence of the presence of C-CTX1 in the common two-banded seabream and the first report of toxicity comparison between liver and muscle from relevant fish species captured in the Canary Islands.

## 1. Introduction

Ciguatoxins (CTXs) are natural marine lipophilic toxins which may cause ciguatera poisoning (CP) in humans. This illness is the most prevalent foodborne disease caused by non-bacterial organisms reported globally (reviewed by [1]). CTXs and other toxic metabolites are produced by a group of microalgae principally of the genus *Gambierdiscus* [2] and *Fukuyoa* [3]. These compounds reach high trophic levels throughout the food chain. Initially, only large carnivores seemed to pose a risk for toxin intake, however it is known that also smaller carnivorous, piscivorous, and herbivorous fish are able to cause this disease [4].

At present, the Canary archipelago is considered an important hotspot for CTXs in Europe [5,6]. Since 2011, the Directorate-General for Fisheries of the Canary Government implemented a protocol to detect CTXs in certain fish species and weights from the authorised points of first sale before reaching the market to protect public health [7]. Additionally, the EuroCigua project, co-funded by the European Food Safety Authority (EFSA) and the University of Las Palmas de Gran Canaria (ULPGC), focused on the characterisation of the risk of CP in Europe. This project highlighted the importance of the Canary Islands for the presence of CTXs in algae and fish, and revealed that amberjack (*Seriola* spp.), dusky grouper (*Epinephelus marginatus*), moray eel (family *Muraenidae*), and the common two-banded seabream (*Diplodus vulgaris*) are relevant species that accumulate toxins in their tissues.

These fish species are of great fishery value in the Canary archipelago [8]. In addition, these species are widely distributed across the globe. Black moray eels (*Muraena helena*) mainly inhabit in the Macaronesia region [9], with a great economic value in the Azores Islands, Madeira, and the Cape Verde archipelago [10]. Dusky groupers can be found in many regions of the Atlantic Ocean and in the Western of the Indian Ocean [11], with a special huge economic interest in the Mediterranean area [12]. Amberjacks are well distributed around the world, be one of the most relevant species in the fishery industry [13,14]. Common two-banded seabream is especially located in the Eastern Atlantic and throughout the Mediterranean Sea and in the Black Sea [15,16]. It is a highly valuable commercial species in different countries such as Turkey and Croatia [17].

Moray eels are demersal and great hunters and are well known to produce CP outbreaks worldwide [18]. The presence of Caribbean ciguatoxin-1 (C-CTX1) [19,20] by liquid chromatography mass spectrometry (LC-MS/MS) in a black moray eel from the Canary Islands [21] has been confirmed. Nevertheless, no reports associated with moray eel consumption have been reported in this area.

Dusky groupers have been studied for their coordinated hunting with moray eels [22]. Therefore, the latter are part of the diet of groupers, as recently reported by Sanchez-Henao et al. [21]. To date, *E. marginatus* has caused four CP outbreaks in the Canary Islands [23]. In dusky groupers from this archipelago, the presence of C-CTX1 has also been analysed by LC-MS/MS [21,24].

The first CP outbreak reported in this archipelago in 2004 was linked to the intake of amberjack flesh [25]. After this event, a total of 11 CP outbreaks have been produced by different species of amberjack, affecting 75 people [23,26]. The presence of C-CTX1 in amberjack captured in the Canary Islands has been confirmed by LC-MS/MS analysis [25,27].

The common two-banded seabream of the *Sparidae* family is an omnivorous fish [28] and *Sarpa salpa*, a member of the same family, has been reported to intake dinoflagellates and produce hallucinatory syndrome in humans [29]. However, these species were not associated with any CP outbreaks.

Despite the worldwide incidence of CP, regulatory measures are necessary in many endemic regions [30]. The official control protocol carried out by the Canary Government, based on the presence or absence of CTX-like toxicity in the muscle of certain fish species, evaluated by cell-based assay (CBA), prevents toxic specimens from being released into the market. The presence of CTXs can only be detected by analytical methods, considering that these toxins do not alter the appearance, smell, or taste of the flesh. Additionally, some authors have observed that the CTXs concentration varies depending on the tissues and fish species; thus, an individual with no detectable CTX-like toxicity in muscle could present CTXs in other tissues [18].

CTXs tend to attach to the cytoplasmic protein of hepatocytes [31] and the liver is therefore a major depot for these toxins [32,33]. Most of what is known about the chemical, pharmacological, and immunological aspects of CTX is from studies which isolated it from the liver of *Gymnothorax* *javanicus*, allowing the scientific community to improve knowledge concerning CTXs [34,35,36]. It is also noted that the viscera of risky fish do not have a market share, as they are considered a high risk to consumers because this toxin is normally most concentrated in visceral tissue [33]. Nonetheless, sport fishermen have viscera available to them, and their consumption could pose a potential risk to public health. Furthermore, these tissues are usually not analysed prior to consumption and, therefore, sport fishery is currently the main cause of CP outbreaks in the Canary Islands.

However, to the best of our knowledge, no studies on CTX detection in the liver of fish species captured in the Canary Islands have been published to date.

The present study deepens the knowledge of the presence of CTX-like toxicity in relevant fish species commonly used for human consumption from this archipelago, comparing CTX levels in the liver and flesh to better evaluate the potential risk of CTXs in viscera to consumers. The study also examined potential factors involved in the levels of CTX-like toxicity found in these fish species (amberjack, dusky grouper, black moray eel, and common two-banded seabream).

## 2. Results

**Figure 1:**
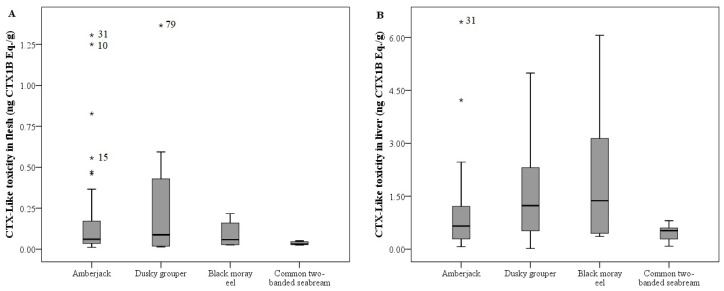
CTX-like toxicity in flesh (**A**) and liver (**B**) by CBA (ng CTX1B Eq·(g tissue)^−1^) according to fish species. The plot represents the interquartile range (Q_3_–Q_1_), and dark line represents the median values. Asterisks (*) indicate outliers and the numbers correspond to specific individuals (Table S1).

**Figure 2:**
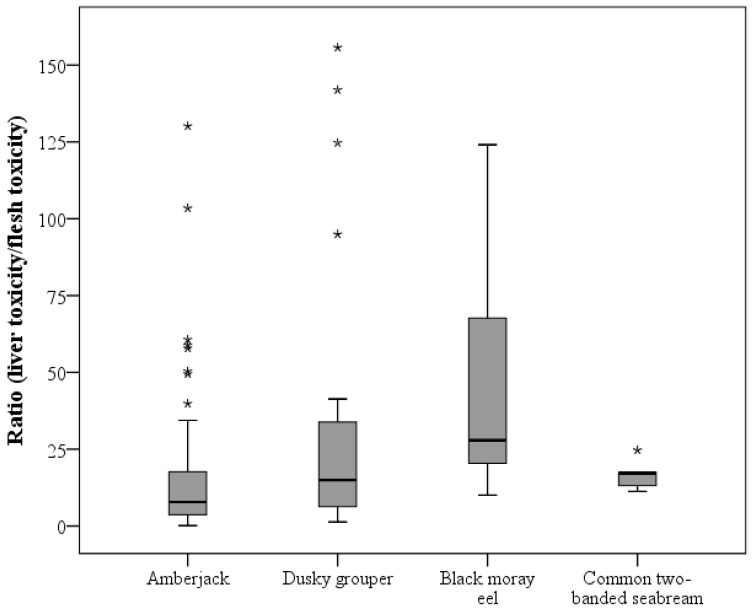
Liver versus flesh CTX concentration ratio according to fish species evaluated by CBA. The plot represents the interquartile range (Q_3_–Q_1_), and dark line represents the median values. Asterisks (*) indicate outliers.

**Figure 3:**
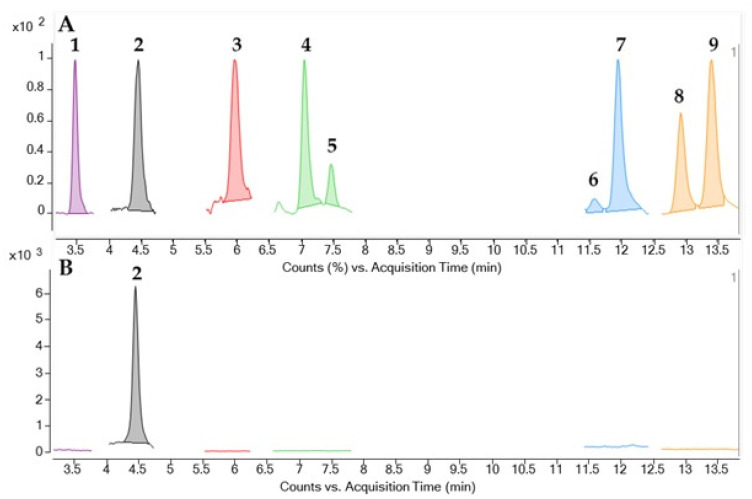
LC-MS/MS chromatogram monitoring CTXs [M + Na]^+^ as precursor and product ion of: (**A**) Reference material of CTXs: CTX1B (1), C-CTX1 (2), C-CTX1-Me (3), 52-*epi*-54-deoxyCTX1B (4), 54-deoxyCTX1B (5), 49-*epi*CTX3C (6), CTX3C (7), CTX4A (8), and CTX4B (9); (**B**) C-CTX1 (2) detected in an amberjack.

A total of 109 specimens were analyzed by CBA to determine the presence of CTXs in the flesh and liver samples. CTX-like toxicity in hepatic tissue was found in 107 fish (98.2%), and 93 of them (85.3%) also showed toxicity in their flesh. The results obtained for both flesh and liver are summarised in Table 1 by fish species, expressed as the range of ng Pacific ciguatoxin-1 (CTX1B) equivalents (Eq.)·(g tissue)^−1^, including the minimum and maximum values reached, mean and median values, and the ratio between CTX concentrations in the liver and flesh. In addition, the distribution of individual toxin concentrations obtained by CBA in the liver and flesh of the different species are represented in a box plot (Figure 1), as well as the liver versus flesh CTX concentration ratio (Figure 2). Furthermore, from all samples, 62 flesh were available to analyse by means of the LC-MS/MS method to identify and quantify the presence of C-CTX1 (Table 2, Figure 3).

### 2.1. Evaluation of the Presence of CTX-like Activity by CBA

#### 2.1.1. In Amberjack

Of the total fish (*n* = 60), all amberjacks with toxic flesh also exhibited hepatic toxicity (*n* = 57); in contrast, two amberjacks with no CTX presence in their flesh displayed toxicity in their livers, and in one individual, no toxicity was found in either tissue. The flesh exhibited mean and median values of 0.165 and 0.061 ng CTX1B Eq·(g flesh)^−1^, respectively, and, by comparison, a mean of 0.953 ng CTX1B Eq·(g liver)^−1^ and a median of 0.655 ng CTX1B Eq·(g liver)^−1^ in the liver (Table 1). The highest CTX concentration observed in the liver of all fish species studied corresponds to an amberjack (number 31), and this individual was the one with the highest concentration of CTX both in the flesh and liver within these species (see Figure 1) and also with the highest concentration of C-CTX1 in flesh measured by LC-MS/MS (0.270 ng·(g flesh)^−1^). In most individuals, the liver showed more toxins than the flesh, with the exception of two specimens. Individual number 10 displayed a CTX-like toxicity of 1.250 ng CTX1B Eq·(g flesh)^−1^ and 0.178 ng CTX1B Eq·(g liver)^−1^. In addition, number 15 showed 0.556 ng CTX1B Eq·(g flesh)^−1^ and 0.195 ng CTX1B Eq·(g liver)^−1^. The livers of the remaining amberjacks (*n* = 55) exhibited more than 17 times the amount of toxins compared to those estimated in the flesh (Table 1). However, as the values were not normally distributed, this average ratio was far from the median (8.39). Among all the fish species studied, these species showed the highest maximum toxicity in the liver (6.439 ng CTX1B Eq·(g liver)^−1^).

#### 2.1.2. In Dusky Grouper

All dusky grouper individuals (*n* = 27) showed toxicity in the liver and 22 displayed toxicities in the flesh. These specimens presented a mean of 0.238 ng CTX1B Eq·(g flesh)^−1^ and a median of 0.088 ng CTX1B Eq·(g flesh)^−1^ (Table 1). Among all the species studied in this research, individual 79 had the highest toxicity level in flesh (1.365 ng CTX1B Eq·(g flesh)^−1^) (Figure 1A, Table 1).

In contrast, for this species, the liver displayed mean and median values of 1.527 and 1.234 ng CTX1B Eq·(g liver)^−1^, respectively. Regarding the ratio between the toxicity levels of both tissues (liver/flesh), all livers were more toxic than the corresponding flesh, with high variability among individuals (from 1- to 156-fold more), which represents a median value of 14.94, greater than the ratio observed in amberjacks.

#### 2.1.3. In Black Moray Eel

All the black moray eels analysed (*n* = 11) displayed toxicity in the liver (1.949 and 1.373 ng CTX1B Eq·(g liver)^−1^, mean and median, respectively), and seven of them also presented CTX in flesh (0.096 and 0.058 ng CTX1B Eq·(g flesh)^−1^, mean and median, respectively). One of the specimens (number 94) was the individual with the highest toxicity level in both the flesh and liver of the black moray eels tested (see Supplementary Table S1). All the livers showed at least ten times the toxicity of flesh, with a mean value of 48.32 and a median of 27.91 (Table 1). One black moray eel specimen (number 98) presented a 124-fold increased toxicity in the liver than in the flesh (see Supplementary Table S1).

Black moray eels showed a high maximum value of toxicity in the liver (6.062 ng CTX1B Eq·(g liver)^−1^), close to that found in amberjack.

#### 2.1.4. In Common Two-Banded Seabream

CTX-like toxicity in liver was observed in 10 out of 11 common two-banded seabreams, and seven of them also displayed toxicity in the flesh. The quantity of CTX found in the flesh showed a mean value of 0.036 and a median value of 0.031 ng CTX1B Eq·(g flesh)^−1^, while the values reached in the liver were 0.454 and 0.525 ng CTX1B Eq·(g liver)^−1^, respectively (Table 1). Every individual exhibited higher CTX concentration in the liver than flesh, with similar mean and median values of the ratios being 16.32 and 17.19, respectively. This species showed the lowest level of toxicity in both types of tissues analysed among all the species studied.

### 2.2. Identification and Quantification of CTX by LC-MS/MS

In the framework of the EuroCigua project, 62 out of the 109 flesh considered in this study were analysed by LC-MS/MS in order to identify and quantify the presence of CTXs in these specimens (Table 2). An example of LC-MS traces showing the detection of C-CTX1 and the absence of additional CTXs is showed in Figure 3. The mixture of CTXs with standard available are compared with the CTXs detected in the sample confirming the detection of C-CTX1 and the absence of other CTXs. The presence of C-CTX1 was confirmed in 30 samples, whereas concentrations below the LOD were obtained for the remaining samples. C-CTX1 was the only CTX analogue identified (Figure 3) in all species evaluated in this study. Twenty-nine of the 57 amberjack flesh samples were investigated using this method, and the levels of C-CTX1 obtained were 0.109 ng·(g flesh)^−1^ as mean and 0.075 ng·(g flesh)^−1^ as median. The average level displayed in the dusky grouper flesh (*n* = 18) was 0.057 ng·(g flesh)^−1^, and the median was 0.030 ng·(g flesh)^−1^. All the toxic flesh samples and one non-toxic from the moray eels were analysed by LC-MS/MS. However, only 2 individuals presented C-CTX1 with the following values: 0.020 and 0.050 ng·(g flesh)^−1^. All the toxic flesh samples from the common two-banded seabream were analysed. The average and median levels in this species were 0.040 and 0.030 ng·(g flesh)^−1^, respectively.

### 2.3. CTX-like Toxicity According to Fish Species by CBA

Although different distributions of concentration, mean, and median values of CTX in flesh were observed between fish species, no statistical difference was found. On the contrary, a significant difference in the liver CTX level between species was found (*p* = 0.025), with black moray eels having the highest mean and median values of CTX in the liver. The common two-banded seabream was the species with the lowest concentration (Figure 1B). A statistically significant difference in the ratio of liver toxin concentration versus flesh between species was found (*p* = 0.014). Amberjack being the species with the lowest median value ratio (8.39), followed by the dusky grouper (14.94), and very closely by seabream (17.19), with the latter having the lowest average ratio (16.32) (Table 1, Figure 2). Moray eels stood out for their great difference between toxin concentrations in the liver and flesh (mean, 48.32 and median, 27.91) (Table 1, Figure 2).

### 2.4. CTX-Like Toxicity According to Fish Weight and Length by CBA

#### 2.4.1. In Amberjack

**Figure 4:**
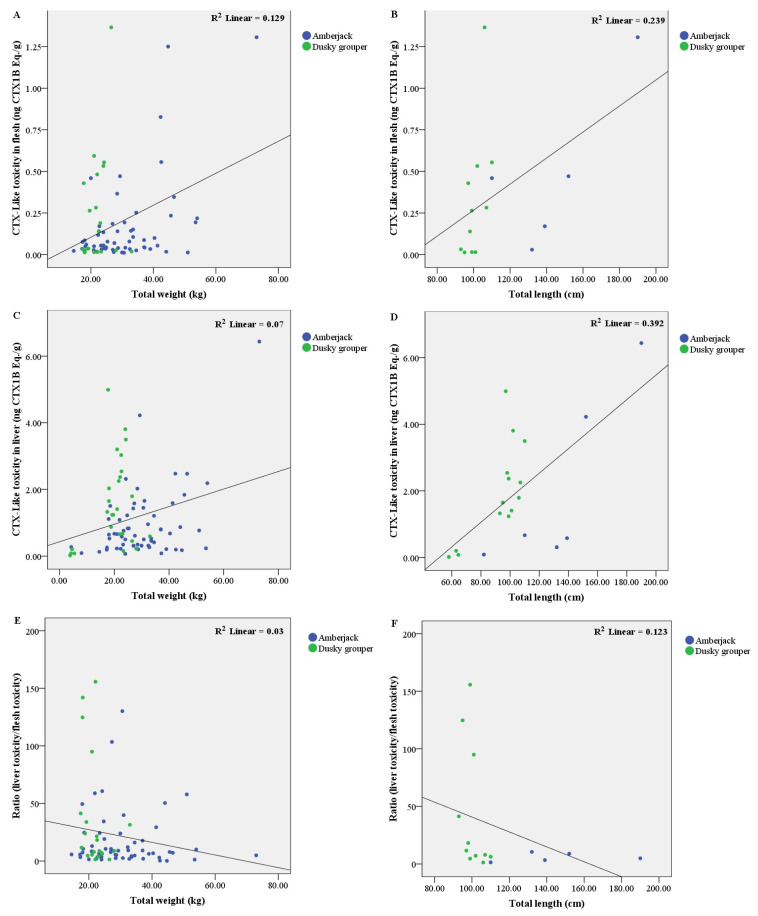
Scatter plots displaying the toxicity results of amberjack and dusky grouper samples, according to the individual morphological features—weight and length. CTX-like toxicity in flesh according to the weight (**A**), and length (**B**). CTX-like toxicity in liver according to the weight (**C**), and length (**D**). Ratio between CTX concentrations of liver and flesh according to the weight (**E**), and length (**F**). The squared correlation coefficient (R2) is also shown.

A positive correlation was observed between liver toxicity level and weight (r = 0.266, *p* = 0.044) and length (r = 0.829, *p* = 0.042) (Figure 4C,D). Likewise, a positive correlation was found between flesh toxicity and weight (r = 0.321, *p* = 0.016) (Figure 4A).

#### 2.4.2. In Dusky Grouper

A negative correlation was detected between the liver/flesh ratio and weight (*r* = −0.435, *p* = 0.049) and length (*r* = −0.579, *p* = 0.062) (Figure 4E,F). In these specimens, the ratio decreased as the individual size increased.

In addition, in these individuals, a strong positive correlation was observed between liver toxicity level and length (*r* = 0.636, *p* = 0.015) (Figure 4D). This positive correlation was also observed between flesh toxicity and length (*r* = 0.624, *p* = 0.040) (Figure 4B).

### 2.5. Influence of Liver Condition

The liver conservation state was recorded at the time of sampling. Most of the studied livers presented different autolysis levels, with the exception of black moray eels, with 50% of the livers showing fresh conditions. Overall, no relationship was observed between the hepatic decomposition level and the variables analysed in this study, either with the CTX-like toxicity or liver/flesh ratio.

## 3. Discussion

### 3.1. Differences in CTX Accumulation in Liver and Flesh

As previously reported by Vernoux et al. [33], it seems that distribution of CTXs in liver versus muscle varies significantly within and between fish species. In almost all of the individuals analysed in the present study (91 out of 93), the liver was more toxic than the flesh. Even when the flesh was non-toxic (*n* = 16), the liver showed a high level of toxicity (14 out of 16). Moray eel livers presented a median nearly 28-fold toxicity compared to flesh which represents the greatest ratio observed among the studied species (see Figure 2). These data are similar to those of previous studies. In 2011, Chan et al. [37], found that the liver of *Gymnothorax* spp. was 9-fold (ranging from 4- to 15-fold) more toxic than flesh. Vernoux et al. [33] analysed the concentration of CTX in the liver and flesh of different fish species, *Muraenidae*, *Serranidae,* and *Carangidae,* among others. They found that the liver presented a minimum of 13-fold higher concentration of CTX than flesh and, in *Gymnothorax funebris*, they observed a maximum of a 114-fold increase in liver than in muscle, comparable with the maximum found in this study in a black moray eel which presented 124 times more toxicity in the liver than in the flesh.

However, the greatest difference in concentrations reached in this study was observed in the dusky grouper (155-fold). In contrast with a paper published by Vernoux et al. [33], in which three individuals of *Epinephelus morio* showed between 7- and 16-fold more toxicity in the liver than in the flesh. In a study published recently by Li et al. [38], the levels of CTXs in the liver from orange-spotted groupers, were between 1.9- and 17-fold higher than that in flesh. In amberjack, the difference between tissues was lower (8-fold as median), slightly lower than the results obtained by Vernoux et al. [33], where the liver of two specimens of *S. dumerili* contained between 10- and 21-fold higher levels of CTXs than flesh. Our results detected more differences between the liver and muscle than previously reported [33,36,38,39]. Of the total individuals analysed in this study, two amberjacks showed muscle with more toxicity than the liver which suggests that the toxin is stored in different ways, depending on the individual and fish species. However, in a high percentage (97.85%) of the fish analysed in the present study, it was observed that the liver contained higher levels of CTX toxicity than the flesh. Thus, in the Canary Islands, the CP official control protocol established by the detection of CTX indicated that the viscera of certain species (*Seriola* spp., *Epinephelus* spp., *Acanthoocybium solandri*, and *Pomatomus saltarix*) cannot be sold independently if the muscle is non-toxic [7]. Interestingly, differences in the course of CP have been observed depending on consumption. Thus, patients who consumed liver showed more severe symptoms than those who ate flesh [40], and some even had a fatal resolution [41].

The high CTX concentrations found in the liver may be due to the fact that it is the target of acute toxicity and the first organ exposed to any compound absorbed [42]. The liver is responsible for the metabolism of xenobiotics among other substances [43] predominantly through cytochrome p450 enzymes (CYPs). It has been observed that CTXs in rat liver [32] induces multiple CYPs (mainly members of CYP2 and CYP4 families), and glutathione S-transferases (GST), which is part of the mechanism of phase II metabolism. It has been reported [44], that CYP in fish liver could oxidise less potent CTXs (CTX4A/B) to more potent CTX (CTX1B). Jiang et al. [45] analysed the liver proteomic profiles of *Cephalopholis argus* and *Gymnothorax undulatus* by observing differences in the proteomics of toxic liver from these two fish species. Besides, they suggested that fish livers develop different resistance strategies to CTX which are species-specific [45,46]. In addition, in experimental studies of dietary CTX exposure in fish [38,47], the liver accumulated a higher portion of CTX in the first hours after a single ingestion of CTX. After these first hours, the concentration of CTX decreased quickly. The muscle requires more time to accumulate CTX, and it is slowly eliminated. These authors suggested that the muscle could act as a target tissue for CTX storage, and that the liver could be a good indicator of recent CTX exposure. This could be the reason why only a few fish in our study (2.15%) showed higher CTX toxicity in muscle than in the liver. The role of flesh as a temporary storage was observed in a study recently published by Sanchez-Henao et al. [48], where a goldfish significantly reduced its CTX-like toxicity after 60 days of feeding with non-toxic food. In the present study, the individuals analysed were supposed to be exposed to CTX for a long time. Positive correlations between size, either the weight or the length, and CTX levels in the liver and flesh were detected in this study. Therefore, the concentration in both tissues continues to increase as the animal increases in size, which is supported by other studies on grouper species [49] and amberjack [5,50]. Other authors have also concluded that fish size and CTX levels may differ between species and regional areas [39,51]. In this regard, a negative correlation between the CTX liver/flesh ratio and size of the specimens was observed in this study, suggesting that as the fish grows, the difference in CTXs between the liver and flesh decreases, which may be explained by the strategy mechanisms developed by the animals to remove toxins from the liver by increasing the efficiency of hepatic detoxification, which could also lead to a greater CTX accumulation in the muscle.

### 3.2. Toxicity Differences among Fish Species

Dusky grouper showed the highest toxicity in the flesh (1.365 ng CTX1B Eq·g^−1^) (Table 1), which is comparable with other studies where groupers were more toxic than other fish species [37,52]. Although the toxicity level of flesh from black moray eels was quite similar to that of the other species studied, the liver was found to be more toxic than the other fish species. In parallel, some livers of black moray eels reached 6.062 ng CTX1B Eq·(g liver)^−1^. In addition, dusky groupers and amberjack flesh were more toxic than the flesh of moray eels and common two-banded seabream. To date, no CP outbreak in the Canary Islands has been linked to the consumption of moray eels, but several cases have been reported in other parts of the world. In these outbreaks, the severity of the disease was related to the intake of viscera, liver, or the head of these fish species (reviewed by [18]). In the Canary archipelago, moray flesh is traditionally consumed fried with the skin; thus, the small portions and the absence of the viscera may be the main reason why there have not been any poisoning cases recorded in the Canary Islands thus far, but other possible species-specific toxicological issues should be considered.

In all the species evaluated, C-CTX1 was the only CTX analogue identified (Figure 3). This CTX profile was similar to the one obtained for samples from the Madeira archipelago [53,54]. The results obtained by CBA showed higher toxicity values than those obtained by LC-MS/MS, as expected, since both methods are not exactly comparable. CBA allowed the detection of CTX-like toxicity and may be caused by the action of multiple CTX analogues and not only C-CTX1.

To the best of our knowledge, this is the first identification of C-CTX1 in common two-banded sea bream (*D. vulgaris*) in the Canary Islands. It is an omnivorous fish, whose diet is based on polychaeta, crustacea, fish, and echinoderms [55,56]. Thus, it has not been considered as a CTX risky fish species, however, it is part of the diet of amberjacks [57], and dusky groupers [58]. Both species are traditionally involved in CP outbreaks. Therefore, our results suggest that *D. vulgaris* can transmit CTX in the Canary archipelago. The lack of CP outbreaks due to the intake of this fish species could be explained by the low toxicity found in flesh (with a maximum of 0.051 ng CTX1B Eq·(g flesh)^−1^) and the usual low dietary exposure of consumers to this fish species. These values are quite different from those obtained by Costa et al., [59] in a zebra seabream (*Diplodus cervines*) captured in the Selvagens Islands (Portugal). In this individual, the toxicity determined by CBA was 0.37 µg CTX1B Eq·(kg flesh)^−1^, and the presence of C-CTX1 was not found. Despite the low toxicity found in flesh of *D. vulgaris*, the liver was found to be 17-fold (median) more toxic than flesh, as found in the rest of the studied species in this study. Although viscera are always discarded by professional fisheries, the present results emphasise the importance of avoiding its consumption by sport fishermen, which are more vulnerable to poisoning as the fish they catch are not subject to official control in this archipelago. Furthermore, the study of the livers of fish individuals from a given area could allow the detection of CP risk situations before they occur.

## 4. Conclusions

The present study investigated the different CTX concentrations found between the liver and flesh of important fish species consumed worldwide (amberjack, dusky grouper, black moray eel, and common two-banded seabream), considering all the consequences that this implies for food safety. Furthermore, the study of the livers of fish individuals from a given area could allow the detection of CP risk situations before they occur. In addition, this study reveals for the first time the presence of C-CTX1 in common two-banded seabream from the Canary Islands, and highlights the sport fishing vulnerability to CP outbreaks.

## 5. Materials and Methods

### 5.1. Study Area

The Canary Islands are a Spanish archipelago of the Macaronesian region located in the North Atlantic Ocean to the south of the European continent and about 100 km east of the coast of Africa. The archipelago consists of eight main volcanic islands and several islets. The islands have a strong fishery tradition [60], and constitute the division 34.1.2 marine fishing area by the Food and Agriculture Organization (FAO) [61].

### 5.2. Fish Sample Collection

The flesh and liver of 109 fish specimens from four different species were analysed in the present study: amberjack (*Seriola* spp.; *n* = 60), dusky grouper (*E. marginatus*; *n* = 27), black moray eel (*M. helena*; *n* = 11), and common two-banded seabream (*D. vulgaris*; *n* = 11). The identification of these fish species was based on morphological characteristics. All individuals were caught in the Canary waters from 2016–2019.

In the Canary Islands, four species of the genus *Seriola* have been described: *Seriola rivoliana*, *Seriola fasciata*, *Seriola dumerili*, and *Seriola carpenteri*. However, due to the absence of molecular techniques to confirm the species level, in this study all amberjacks have been identified only with the genus *Seriola*.

The specimens were obtained from different sampling sources. Most of them were provided by the official control protocol of the Canary Government to prevent CP (*n* = 79; species: amberjack and dusky grouper). It is important to highlight that most individuals from the official monitoring were ciguatoxic, and they were selected to achieve the objectives proposed by this study. The remaining individuals were sampled in the framework of the EuroCigua project (GP/EFSA/AFSCO/2015/03) (*n* = 29; all studied species) from local fisheries or authorised first sale points on different islands provided by sport fishermen (*n* = 1), and one black moray eel was recovered from the stomach content of a dusky grouper.

Details regarding the capture island, year, and season were taken into account when analysing these individuals (see Supplementary Table S1).

### 5.3. Muscle Sample Preparation and CTX Extraction

Each fish was kept frozen until processing. Muscle and liver samples were collected from each individual, and when possible, the total length (nearest mm), and weight (nearest g) were recorded (Table 3). Samples were extracted according to the protocol described in [62], with slight modifications according to laboratory needs [5].

For the matrix effect, the protocol employed was that described by [63] with minor modifications. Initially, fish flesh was homogenised, after which 10 g was cooked at 70 °C for 10 min in a water bath. When the sample reached room temperature (22 °C), 20 mL of acetone was added, mixed with an Ultraturrax blender, and centrifuged at 3000× *g* for 10 min at 4 °C, and the final step was repeated twice and both supernatants were pooled. The resulting acetone was filtered through a 0.45 µm PTFE filter and dried on a rotary evaporator at 55 °C. The dried extract obtained was re-suspended twice in Milli Q water with diethyl ether (DEE). Both DEE phases were pooled and evaporated with a rotary evaporator at 55 °C for a subsequent dissolution with methanol:water (8:2) and partitioned twice with *n*-hexane. The *n*-hexane was discarded, and the methanol phase was evaporated to dryness under a N_2_ current at 40 °C. The final residue was dissolved in 4 mL of methanol and kept at −20 °C to be used in the cytotoxicity assay.

### 5.4. Liver Sample Preparation and Extraction of CTX

At the time of sampling, the conservation state of the liver was recorded according to its morphological features and was graded as 1 (very fresh) to 5 (very advanced autolysis) (Table S1), given that the liver is a susceptible organ which deteriorates quickly after death.

When possible, 10 g of liver were obtained and cooked at 70 °C for 10 min in a water bath. However, for some samples, the weight of the liver was not sufficient to fulfil the protocol and ranged from 1.2–8.9 g. The protocol was performed as previously described above for the flesh, with slight differences. The centrifugation time was 15 min, and three partitions of *n*-hexane were conducted.

### 5.5. Cytotoxicity CBA (Neuro-2a-MTT)

Neuro-2a neuroblastoma cells (cell line: CCL131) were purchased from the American Type Culture Collection (ATCC, LGC Standards S.L.U., Barcelona, Spain). Cells were grown and maintained in Roswell Park Memorial Institute (RPMI)-1640 medium containing 5–10% fetal bovine serum at 37 °C in a 5% CO_2_ atmosphere. The Pacific type 1 CTX standard (STD) (named CTX1B) (R.J. Lewis, Queensland University, Brisbane, Queensland, Australia) was used to assess CTX-like toxicity.

The cytotoxicity assay was conducted as previously described [5] with minor modification in cell density (at 40,000 cells per well).

The assessment of the matrix effect on the Neuro-2a assay was performed with a dose of 200 mg tissue equivalents (TE)/mL from different species. Half dilutions of this concentration were conducted to expose cells with or without ouabain/veratridine (*O*/*V*) pretreatment. At the first dose, several samples showed interference with the assay, whereas at a concentration of 100 mg TE/mL this effect disappear in flesh extracts. Regarding the livers, it was observed that concentrations higher than 50 mg TE/mL produced matrix effects which may mask CTX-like toxicity. Regarding hepatic samples from common two-banded seabream, matrix interferences were observed even at 50 mg TE/mL, but not at 25 mg TE/mL (see Supplementary Figure S1).

Flesh and liver extracts were tested at a maximum concentration of 200 and 50 mg TE of matrix/mL, respectively, to avoid matrix effects on cells. Tissues from the same individuals were assessed under the same methodological conditions. The limit of detection (LOD) and limit of quantification (LOQ) of CBA were set at the level of CTX1B STD that caused 20% inhibition of cell viability (IC_20_), considering the concentration of extracts used for the analysis.

The LOD/LOQ mean values obtained for both tissues were 0.015 ng CTX1B Eq·(g flesh)^−1^ (min.: 0.004 and max.: 0.074, depending on the particularity of the tissue) and 0.043 ng CTX1B Eq·(g liver)^−1^ (min.: 0.015 and max.: 0.118, depending on the particularity of the tissue).

Toxic content in fish analysed for CTX-like toxicity by CBA was expressed in ng CTX1B Eq·(g tissue)^−1^.

### 5.6. CTX Identification by LC-MS/MS

In order to confirm the presence of CTX in the individuals analysed, 62 flesh samples from all the species involved in this study were analysed by LC-MS/MS in the framework of the EuroCigua project.

CTX1B (4466 ng/mL) and a mixture of P-CTXs containing: CTX1B, 52-*epi*-54-deoxyCTX1B (previously named P-CTX2) [20,64], 54-deoxyCTX1B (previously named P-CTX3) [20,64], 49-*epi*CTX3C, CTX3C, CTX4A and CTX4B were kindly provided by Prof. Takeshi Yasumoto (Japan Food Research Laboratories, Tokyo, Japan). The C-CTX1 standard was kindly provided by Dr. Robert W. Dickey (University of Texas, Austin, TX, USA) via Dr. Ronald Manger (Fred Hutchinson Cancer Research Center, Seattle, DC, USA). A laboratory reference material containing C-CTX1-Me was obtained by isolation and purification of this toxin from naturally contaminated fish tissue used from previous work carried out by some of the authors of this study [65].

Sample pretreatment for LC-MS/MS analyses was carried out following the conditions described by [24]. Briefly, 15 g of fish tissue were extracted twice with acetone (45 mL). Acetone layers were combined and evaporated to an aqueous residue which was extracted twice with DEE (15 mL). The combined DEE layers were evaporated to a solid residue. Solid residue was dissolved in 90% methanol/water (*v*/*v*) (4.5 mL) and defatted with *n*-hexane (9 mL). The methanolic layer was evaporated to dryness and submitted to the purification step. The solid residue was dissolved in ethyl acetate (2 mL) and loaded in a Florisil SPE cartridge (500 mg, 3 mL) previously conditioned with ethyl acetate (3 mL). The cartridge was washed with ethyl acetate (3 mL) and the CTXs were eluted with ethyl acetate/methanol 9/1 (*v*/*v*) (5 mL). The toxic eluate from Florisil SPE was evaporated to a solid residue, dissolved in 60% methanol/water (*v*/*v*) (2 mL) and loaded in a C18 SPE cartridge (500 mg, 3 mL) previously conditioned with 60% methanol/water (*v*/*v*) (3 mL). The C18 SPE cartridge was washed with 60% methanol/water (*v*/*v*) (3 mL) and the CTXs were eluted with 90% methanol/water (*v*/*v*) (5 mL). The toxic eluate containing the CTXs was evaporated to dryness, and the solid residue was dissolved in methanol LC-MS grade (0.5 mL, 30 g flesh/mL), and filtered through 0.22 µm prior the LC-MS/MS analyses.

LC-MS/MS analyses were performed using an Agilent 1290 Infinity Liquid Chromatography system coupled to an Agilent 6495 triple quad iFunnel (Agilent Technologies, Waldbronn, Germany) according to the method described by [24].

Chromatographic separation was carried out on a C18 column (Poroshell 120-EC-C18, 3.0 × 50 mm, 2.7 µm, Agilent, Santa Clara, CA, USA) set at 40 °C. Mobile phases consisted of 0.1% formic acid and 5 mM ammonium formate in water (A) and methanol (B). The injection volume was 1 µL and the flow rate was 0.4 mL/min. CTXs were separated using a gradient of mobile phases starting at 78% B, increasing to 88% B in 10 min, keeping for 5 min, and increasing from 15.01 min to 100% B for 3 min. The gradient of the mobile phase returned to the initial conditions of 78% B in 18.01 min, equilibrating the column 4 min prior to the next injection.

CTX sodium adduct [M + Na]^+^ was monitored in positive ionisation mode as the precursor and product ion using the multiple reaction monitoring (MRM) mode. The collision energy (CE) and collision acceleration voltage (CAV) were set at 40 eV and 4 eV, respectively, for all CTXs monitored. CTXs with the reference material available were monitored as follows: CTX1B (*m*/*z* 1133.6 -> *m*/*z* 1133.6), C-CTX1 (*m*/*z* 1163.7 -> *m*/*z* 1163.7), C-CTX1-Me (*m*/*z* 1177.6 -> *m*/*z* 1177.6), 52-*epi*-54-deoxyCTX1B/54-deoxyCTX1B (*m*/*z* 1117.6 -> *m*/*z* 1117.6), 49-*epi*CTX3C/CTX3C (*m*/*z* 1045.6 -> *m*/*z* 1045.6), and CTX4A/CTX4B (*m*/*z* 1083.6 -> *m*/*z* 1083.6).

The ion source and interface settings were as follows: gas flow, 15 L/min; gas temperature, 290 °C; sheath gas flow, 12 L/min; sheath gas temperature, 400 °C; nebuliser pressure, 50 psi; fragmentor potential, 380 V; capillary voltage, 5000 V; and nozzle voltage, 300 V.

The LOD and LOQ for CTX1B were 0.004 and 0.015 ng·g^−1^, respectively. The limited amount available for the C-CTX1 standard did not allow the performance of full calibration studies required for proper quantitation. Therefore, the quantitation of C-CTX1 in the contaminated samples was carried out using CTX1B for calibration, selecting 0.45–27.88 ng CTX1B·mL^−1^ as the range, *n* = 5; equivalent to 0.015–0.929 ng CTX1B·g^−1^. CTX1B Eq. were transformed into C-CTX1 Eq. by using a correction factor obtained from the quantitation of the standard of C-CTX1 in the CTX1B calibration curve as described by [24].

### 5.7. Statistical Analysis

Data analysis was conducted using PASW Statistics software version 18.0 for Windows (SPSS Inc., Chicago, IL, USA).

Normality of data was evaluated using the Kolmogorov-Smirnov test or Shapiro-Wilk test, as appropriate. Due to the absence of normality, non-parametric tests such as the Mann-Whitney U and Kruskal-Wallis tests were used. Chi-square and exact Fisher tests were conducted to compare the percentages of nominal variables. The non-parametric Spearman’s rank order test was used to compare numeric variables. As usual, a *p*-value ≤ 0.05, was considered statistically significant.

## Figures and Tables

**Table 1:** Estimated concentration of ciguatoxins (CTXs) content according to CTX-like toxicity obtained by cell-based assay (CBA) in flesh and liver, and the ratio between CTXs concentration of liver and flesh. Data expressed as mean ± standard deviation (SD), median, minimum, and maximum values in ng Pacific ciguatoxin-1 (CTX1B) equivalents (Eq.)·(g tissue)^−1^.

Fish Studied		Amberjack (*Seriola* spp.) *n*^a^ = 60	Dusky Grouper (*E. marginatus)* *n*^a^ = 27	Black Moray Eel (*M. helena)* *n*^a^ = 11	Common Two-Banded Seabream (*D. vulgaris*) *n*^a^ = 11	Total *n*^a^ = 109
ng CTX1B Eq·(g flesh)^−1^	*n* ^b^	57	22	7	7	93
	Mean ± SD	0.165 ± 0.264	0.238 ± 0.325	0.096 ± 0.081	0.036 ± 0.011	
	Median	0.061	0.088	0.058	0.031	
	Minimum	0.011	0.013	0.026	0.024	
	Maximum	1.306	1.365	0.217	0.051	
ng CTX1B Eq·(g liver)^−1^	*n* ^b^	59	27	11	10	107
	Mean ± SD	0.953 ± 1.066	1.527 ± 1.317	1.949 ± 1.816	0.454 ± 0.223	
	Median	0.655	1.234	1.373	0.525	
	Minimum	0.069	0.020	0.361	0.084	
	Maximum	6.439	4.991	6.062	0.808	
Ratio (liver toxicity/flesh toxicity)	*n* ^b^	55	22	7	7	91
	Mean ± SD	17.84 ± 25.05	35.51 ± 47.63	48.32 ± 41.55	16.32 ± 4.49	
	Median	8.39	14.94	27.91	17.19	
	Minimum	1.21	1.31	10.06	11.25	
	Maximum	130.15	155.70	124.09	24.69	

*n*^a^ = Sample size. *n*^b^ = Individuals with CTX-like toxicity.

**Table 2:** Level of Caribbean ciguatoxin-1 (C-CTX1) from the flesh tissue of the individuals analysed by liquid chromatography mass spectrometry (LC-MS/MS). C-CTX1 levels were quantified using CTX1B calibration curve due to unavailability of C-CTX1 reference material. Data expressed as mean ± standard deviation (SD), median, minimum, and maximum values.

Fish Studied		Amberjack (*Seriola* spp.) *n* = 29	Dusky Grouper (*E. marginatus*) *n* = 18	Black Moray Eel (*M. helena*) *n* = 8	Common Two-Banded Seabream (*D. vulgaris*) *n* = 7	Total *n* = 62
ng·(g flesh)^−1^ by LC-MS/MS	C-CTX1 confirmed ^a^	8	15	2	5	30
	Mean ± SD	0.109 ± 0.091	0.057 ± 0.059	0.035 ± 0.021	0.040 ± 0.017	
	Median	0.075	0.030	0.035	0.030	
	Minimum	0.020	0.018	0.020	0.030	
	Maximum	0.270	0.240	0.050	0.070	

*n* = Sample size. ^a^ = Individuals with presence of C-CTX1 in flesh confirmed by LC-MS/MS.

**Table 3:** Information regarding the individual weight and length of all the fish species studied. Data expressed as mean ± standard deviation (SD), median, minimum, and maximum values.

Fish Studied		Amberjack *n* = 60	Dusky Grouper *n* = 27	Black Moray Eel *n* = 11	Common Two-Banded Seabream *n* = 11
Individual weight ^a^	Mean ± SD	29.97 ± 12.41	19.53 ± 7.43	1.24 ± 0.86	0.46 ± 0.16
	Median	28.50	21.30	0.98	0.48
	Minimum	3.53	3.80	0.41	0.15
	Maximum	73.00	33.00	2.81	0.71
Individual length ^b^	Mean ± SD	124.43 ± 42.33	92.32 ± 17.20	76.25 ± 17.24	28.17 ± 3.17
	Median	132.00	98.50	74.00	29.50
	Minimum	66.00	58.00	56.90	21.00
	Maximum	190.00	110.00	110.00	32.00

^a^ Total weight in kg. ^b^ Total length in cm. *n* = Sample size.

## Supplementary Materials

The following supporting information can be downloaded at: https://www.mdpi.com/article/10.3390/toxins14010046/s1, Figure S1: Representative matrix effect dose-response curves obtained by CBA with an amberjack flesh sample (A), an amberjack liver sample (B), and a common two-banded seabream liver sample (C). Red line indicates the limit of exposure (mg tissue equivalent (TE)/mL) for matrix interferences; Table S1: Information details regarding the capture—island, year, and season—morphological features—weight, length, and liver conservation state—and toxicity results obtained by cell-based assay in flesh and liver of each specimen analysed in this study.
