# Supplementary material for: Ciguatoxin Detection in Flesh and Liver of Relevant Fish Species from the Canary Islands
Source: Toxins (Basel). 2022 Jan 9;14(1):46. doi: 10.3390/toxins14010046 (PMC8781511; doi:10.3390/toxins14010046)
Supplement: Supplementary file 1 [file toxins-14-00046-s001.zip › toxins-1503371-supplementary.pdf]

toxins

MDPI

Supplementary Materials: Ciguatoxin Detection in Flesh and Liver of Relevant Fish Species from the Canary Islands

María José Ramos-Sosa, Natalia García-Álvarez, Andres Sanchez-Henao, Freddy Silva Sergent, Daniel Padilla, Pablo Estévez, María José Caballero, José Luís Martín-Barrasa, Ana Gago-Martínez, Jorge Diogène and Fernando Real

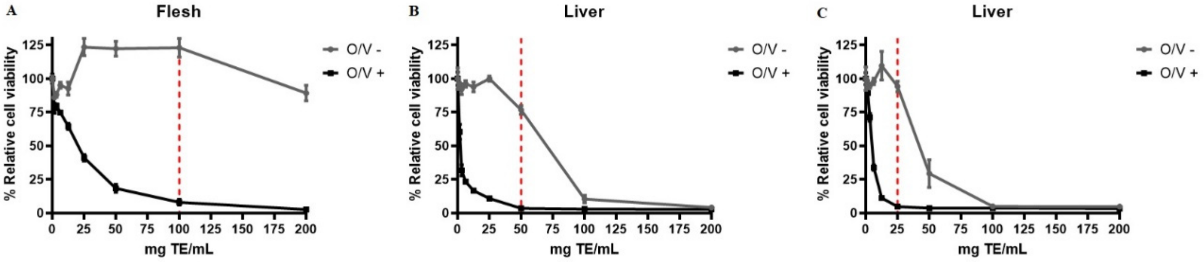

Figure S1. Representative matrix effect dose-response curves obtained by CBA with an amberjack flesh sample (**A**), an amberjack liver sample (**B**), and a common two-banded seabream liver sample (**C**). Red line indicates the limit of exposure (mg tissue equivalent (TE)/mL) for matrix interferences.

Table S1. Information details regarding the capture - island, year, and season - morphological features – weight, length, and liver conservation state – and toxicity results obtained by cell-based assay in flesh and liver of each specimen analysed in this study.

**Reference**	**Species**	**Capture Island**	**Capture Year**	**Capture Season**[a]	**Weight**[b]	**Lenght**[c]	**Liver Conservation State**[d]	**ng CTX1B equivalents (g flesh)$^{-1}$**	**LOD/LOQ**	**ng CTX1B equivalents (g liver)$^{-1}$**	**LOD/LOQ**	**Ratio**[e]
1	Amberjack	Fuerteventura	2016	Warm	54.00		3	0.2182	0.0091	2.1854	0.0428	10.02
2	Amberjack	Fuerteventura	2016	Warm	33.50		5	0.1511	0.0235	0.5238	0.0556	3.47
3	Amberjack	Lanzarote	2016	Warm	24.00			0.1340	0.0178	0.4935	0.0178	3.68
4	Amberjack	Lanzarote	2016	Warm	27.30		4	0.0153	0.0091	1.5774	0.0556	103.44
5	Amberjack	Fuerteventura	2016	Warm	24.90		5	0.0432	0.0164	0.8268	0.0633	19.14
6	Amberjack	Lanzarote	2016	Warm	23.20		4	0.0546	0.0204	0.3414	0.0654	6.26
7	Amberjack	Lanzarote	2016	Warm	27.00	132.0	3	0.0294	0.0044	0.3091	0.0633	10.51
8	Amberjack	Lanzarote	2016	Warm	21.00		3	0.0504	0.0198	0.6551	0.0633	13.00
9	Amberjack	Lanzarote	2016	Warm	45.60			0.2343	0.0178	1.8353	0.0178	7.83
10	Amberjack	Fuerteventura	2016	Warm	44.70		5	1.2500	0.0735	0.1778	0.1176	0.14
11	Amberjack	Lanzarote	2016	Warm	20.90		4	0.0274	0.0164	0.2299	0.0327	8.39
12	Amberjack	Lanzarote	2016	Warm	30.75		3	0.1933	0.0204	0.4954	0.0654	2.56
13	Amberjack	Lanzarote	2016	Warm	22.70	139.0	4	0.1705	0.0098	0.5776	0.0178	3.39
14	Amberjack	Lanzarote	2016	Warm	28.50		4	0.1402	0.0098	0.3414	0.0178	2.44
15	Amberjack	Lanzarote	2016	Warm	42.50		4	0.5564	0.0327	0.1953	0.0657	0.35
16	Amberjack	Fuerteventura	2016	Warm	24.00			0.0553	0.0198	0.0685	0.0317	1.24
17	Amberjack	Fuerteventura	2016	Warm	32.80		4	0.1429	0.0235	0.2643	0.0317	1.85
18	Amberjack	Lanzarote	2016	Warm	33.50		4	0.1061	0.0198	0.4514	0.0633	4.25
19	Amberjack	Lanzarote	2016	Warm	41.30		3	0.0541	0.0198	1.5858	0.0633	29.31
20	Amberjack	Lanzarote	2016	Warm	20.00	110.0	4	0.4595	0.0235	0.6641	0.1176	1.45
21	Amberjack	Lanzarote	2017	Cold	29.95		4	0.0130	0.0044	0.3110	0.0178	23.92
22	Amberjack	Lanzarote	2017	Cold	34.50		5	0.0254	0.0107	0.4075	0.0428	16.04
23	Amberjack	Lanzarote	2017	Warm	42.30			0.8267	0.0180	2.4696	0.0310	2.99
24	Amberjack	Lanzarote	2017	Warm	17.20		2	0.0346	0.0044	0.1917	0.0178	5.54
25	Amberjack	Fuerteventura	2017	Warm	37.00		5	0.0873	0.0268	0.7999	0.0588	9.16
26	Amberjack	Fuerteventura	2017	Warm	21.90		2	0.0184	0.0055	1.0826	0.0588	58.84
27	Amberjack	Tenerife	2017	Warm	51.00		5	0.0132	0.0091	0.7636	0.1176	57.85
28	Amberjack	Lanzarote	2017	Warm	25.30		4	0.0783	0.0308	0.8266	0.0214	10.56
29	Amberjack	Lanzarote	2017	Warm	31.00		5	0.0415	0.0171	1.6538	0.0428	39.85
30	Amberjack	La Gomera	2017	Warm	39.00			0.0336	0.0051	0.2104	0.0442	6.26

31	Amberjack	Tenerife	2017	Warm	73.00	190.0		1.3061	0.0070	6.4390	0.0290	4.93
32	Amberjack	Lanzarote	2017	Warm	32.30			0.0788	0.0107	0.9505	0.0214	12.06
33	Amberjack	Fuerteventura	2017	Warm	24.70			0.0354	0.0086	1.2168	0.0214	34.37
34	Amberjack	La Palma	2017	Warm	14.50		4	0.0225	0.0170	0.1285	0.0504	5.71
35	Amberjack	Fuerteventura	2017	Warm				0.0550	0.0459	0.7289	0.0588	13.25
36	Amberjack	Fuerteventura	2017	Warm	24.20			0.0381	0.0086	2.3118	0.0214	60.68
37	Amberjack	Fuerteventura	2017	Warm	17.90			0.0224	0.0086	1.1085	0.0428	49.49
38	Amberjack	Fuerteventura	2017	Warm	23.40			0.0311	0.0077	0.7566	0.0154	24.33
39	Amberjack	Fuerteventura	2017	Warm	17.30			0.0759	0.0077	0.2534	0.0154	3.34
40	Amberjack	Fuerteventura	2017	Warm	18.20			0.0493	0.0077	0.5214	0.0154	10.58
41	Amberjack	Fuerteventura	2017	Warm	18.00			0.0861	0.0158	0.6399	0.0252	7.44
42	Amberjack	Fuerteventura	2017	Warm	18.50			0.0607	0.0077	1.5000	0.0154	24.71
43	Amberjack	Fuerteventura	2017	Warm	27.50			0.0695	0.0138	0.6067	0.0221	8.73
44	Amberjack	Tenerife	2017	Warm	44.10			0.0172	0.0158	0.8660	0.0252	50.35
45	Amberjack	Tenerife	2017	Warm	30.60			0.0111	0.0062	1.4447	0.0252	130.15
46	Amberjack	Tenerife	2017	Warm	28.40			0.3660	0.0210	2.0210	0.0350	5.52
47	Amberjack	Tenerife	2017	Warm	28.70			0.0394	0.0294	0.2044	0.0186	5.19
48	Amberjack	Fuerteventura	2017	Warm	37.20			0.0422	0.0077	0.0829	0.0309	1.96
49	Amberjack	Lanzarote	2017	Warm	29.30	152		0.4710	0.0178	4.2230	0.0178	8.97
50	Amberjack	Gran Canaria	2018	Cold	3.53	66.0		<LOQ	0.0082	<LOQ	0.0327	
51	Amberjack	Gran Canaria	2018	Warm	53.50			0.1942	0.0158	0.2353	0.0252	1.21
52	Amberjack	Tenerife	2018	Warm	22.30			0.1176	0.0185	0.2194	0.0252	1.87
53	Amberjack	Lanzarote	2018	Warm	46.60			0.3458	0.0143	2.4671	0.0229	7.13
54	Amberjack	Lanzarote	2018	Warm	37.00			0.0447	0.0111	0.7892	0.0145	17.66
55	Amberjack	Fuerteventura	2018	Warm	26.90			0.1854	0.0174	1.4252	0.0278	7.69
56	Amberjack	Gran Canaria	2018	Warm	40.35			0.0999	0.0102	0.6734	0.0155	6.74
57	Amberjack	Gran Canaria	2018	Warm	32.45			0.0335	0.0102	0.3110	0.0278	9.28
58	Amberjack	Gran Canaria	2018	Warm	34.50			0.2522	0.0102	1.2048	0.0309	4.78
59	Amberjack	Lanzarote	2018	Warm	4.25		2	<LOQ	0.0103	0.2680	0.0795	
60	Amberjack	El Hierro	2019	Warm	8.00	82.0	5	<LOQ	0.0252	0.0910	0.0371	
61	Dusky grouper	Lanzarote	2016	Warm	28.00		4	0.0253	0.0185	0.2256	0.0442	8.92
62	Dusky grouper	Lanzarote	2016	Warm	23.00		4	0.1898	0.0315	0.6673	0.0457	3.52
63	Dusky grouper	El Hierro	2016	Warm	24.00	102.0		0.5326	0.0190	3.8056	0.0310	7.15
64	Dusky grouper	Lanzarote	2016	Warm	19.60	99.0	5	0.2641	0.0336	1.2337	0.0165	4.67
65	Dusky grouper	Fuerteventura	2016	Warm			4	0.0332	0.0068	0.8896	0.1176	26.80
66	Dusky grouper	Tenerife	2017	Cold	22.60	98.0		0.1391	0.0110	2.5380	0.0170	18.25
67	Dusky grouper	Tenerife	2017	Cold	21.60	107.0		0.2822	0.0178	2.2500	0.0178	7.97

68	Dusky grouper	Lanzarote	2017	Cold	23.30		2	0.0177	0.0080	0.1548	0.0327	8.74
69	Dusky grouper	Lanzarote	2017	Warm	22.00		4	0.4820	0.0209	0.6551	0.0327	1.36
70	Dusky grouper	Lanzarote	2017	Warm	22.50		4	0.1409	0.0268	3.0288	0.0428	21.50
71	Dusky grouper	Fuerteventura	2017	Warm	17.70	97.0		0.4291	0.0178	4.9910	0.0178	11.63
72	Dusky grouper	Lanzarote	2017	Warm	18.00	95.0	4	0.0132	0.0091	1.6460	0.1176	124.70
73	Dusky grouper	Tenerife	2017	Warm	24.20	110.0		0.5544	0.0178	3.4942	0.0178	6.30
74	Dusky grouper	Tenerife	2017	Warm	21.00	101.0	2	0.0148	0.0104	1.4051	0.0330	94.94
75	Dusky grouper	Lanzarote	2017	Warm	17.40	93.0		0.0320	0.0280	1.3225	0.0221	41.33
76	Dusky grouper	Lanzarote	2017	Warm	22.10	99		0.0152	0.0056	2.3667	0.0330	155.70
77	Dusky grouper	Fuerteventura	2017	Warm	18.05			0.0143	0.0086	2.0294	0.0428	141.92
78	Dusky grouper	Tenerife	2017	Warm	33.00			0.0187	0.0060	0.5879	0.0588	31.44
79	Dusky grouper	Lanzarote	2018	Cold	26.50	106.0		1.3654	0.0178	1.7912	0.0178	1.31
80	Dusky grouper	Gran Canaria	2018	Cold	3.80	58.0		<LOQ	0.0041	0.0200	0.0178	
81	Dusky grouper	Lanzarote	2018	Warm	21.00			0.5932	0.0091	3.2016	0.0290	5.40
82	Dusky grouper	Lanzarote	2018	Warm	18.80			0.0364	0.0178	0.8691	0.1176	23.88
83	Dusky grouper	Lanzarote	2018	Warm	19.20			0.0364	0.0178	1.2321	0.0588	33.85
84	Dusky grouper	Lanzarote	2018	Warm	4.52	63.0	3	<LOQ	0.0041	0.2037	0.0330	
85	Dusky grouper	Lanzarote	2018	Warm	5.40		4	<LOQ	0.0041	0.0811	0.0330	
86	Dusky grouper	Lanzarote	2019	Cold	26.50		4	<LOQ	0.0170	0.4471	0.0398	
87	Dusky grouper	El Hierro	2019	Warm	4.13	64.5	3	<LOQ	0.0083	0.0822	0.0165	
88	Black moray eel	Lanzarote	2017	Warm	1.03	82.0		0.0260	0.0201	2.1357	0.0624	82.14
89	Black moray eel	Tenerife	2018	Cold	0.87	70.0	3	0.0290	0.0134	0.6611	0.0486	22.80
90	Black moray eel	La Gomera	2018	Warm	1.47	80.5		0.1364	0.0143	1.3725	0.0457	10.06
91	Black moray eel	Lanzarote	2018	Warm	1.65	83.0	2	0.1820	0.0252	3.2776	0.0698	18.01
92	Black moray eel	La Gomera	2018	Warm	0.55	58.0	1	<LOQ	0.0082	0.4308	0.0654	
93	Black moray eel	El Hierro	2018	Warm	0.41	56.9	2	0.0577	0.0085	3.0712	0.0698	53.23
94	Black moray eel	El Hierro	2018	Warm	2.81	100.3		0.2172	0.0143	6.0622	0.0428	27.91
95	Black moray eel	Gran Canaria	2018	Warm	2.76	110.0		<LOQ	0.0061	0.3610	0.0244	
96	Black moray eel	El Hierro	2018	Warm	0.56	61.0	3	<LOQ	0.0164	0.4641	0.0654	
97	Black moray eel	Tenerife	2018	Warm	0.98	74.0	3	<LOQ	0.0164	0.3999	0.0654	
98	Black moray eel	La Palma	2018	Warm	0.52	63.0		0.0258	0.0041	3.2016	0.0165	124.09
99	Common two-banded sea bream	Gran Canaria	2019	Warm	0.23	24.50	5	<LOQ	0.0069	<LOQ	0.0278	
100	Common two-banded sea bream	Gran Canaria	2019	Warm	0.15	21.00	1	<LOQ	0.0087	0.0841	0.0349	
101	Common two-banded sea bream	El Hierro	2019	Warm	0.48	30.4	5	<LOQ	0.0069	0.2873	0.0654	

102	Common two-banded sea bream	El Hierro	2019	Warm	0.38	26.0	5	0.0309	0.0153	0.5313	0.0613	17.19
103	Common two-banded sea bream	El Hierro	2019	Warm	0.46	29.0	5	0.0301	0.0123	0.5190	0.0613	17.24
104	Common two-banded sea bream	El Hierro	2019	Warm	0.50	28.0	4	0.0459	0.0153	0.8075	0.0613	17.58
105	Common two-banded sea bream	El Hierro	2019	Warm	0.51	30.0	5	<LOQ	0.0116	0.2114	0.0556	
106	Common two-banded sea bream	El Hierro	2019	Warm	0.55	30.0	5	0.0242	0.0153	0.5968	0.0613	24.69
107	Common two-banded sea bream	El Hierro	2019	Warm	0.47	29.5	5	0.0245	0.0123	0.2969	0.0613	12.12
108	Common two-banded sea bream	El Hierro	2019	Warm	0.71	32.0	5	0.0507	0.0123	0.5697	0.0613	11.25
109	Common two-banded sea bream	El Hierro	2019	Warm	0.58	29.5	5	0.0445	0.0069	0.6310	0.0556	14.19

[a] Capture season: «Cold»: January – April; «Warm»: May – December.

[b] Total weight in kg.

[c] Total length in cm.

[d] 1, very fresh; 2, fresh; 3, moderate autolysis; 4, advanced autolysis; 5, very advanced autolysis.

[e] Liver toxicity/flesh toxicity.
